# Adaptation to dislodgement risk on wave-swept rocky shores in the snail *Littorina saxatilis*

**DOI:** 10.1371/journal.pone.0186901

**Published:** 2017-10-23

**Authors:** Guénolé Le Pennec, Roger K. Butlin, Per R. Jonsson, Ann I. Larsson, Jessica Lindborg, Erik Bergström, Anja M. Westram, Kerstin Johannesson

**Affiliations:** 1 Department of Marine Sciences, Tjärnö, University of Gothenburg, Strömstad, Sweden; 2 Department of Animal and Plant Sciences, University of Sheffield, Sheffield, United Kingdom; University of California, UNITED STATES

## Abstract

The periwinkle *Littorina saxatilis* has repeatedly evolved both a small, fragile and globose "wave ecotype" confined to wave-swept shores and a large, robust and elongated "crab ecotype" found in nearby crab-rich but less-exposed shores. This phenotypic divergence is assumed to reflect, in some part, local adaptation to wave exposure, but this hypothesis has received incomplete experimental testing. Here, we report a test of the prediction that the wave ecotype has a higher capacity to resist water flow than the crab ecotype. We sampled snails along a crab-wave transect and measured their resistance to dislodgement in a high-speed water flume with water speeds that match those of breaking waves in a range of relevant field conditions. Snails from the wave environment were consistently more resistant to water flow than snails from the crab environment and high resistance was positively correlated with the surface area of the foot and the area of the outer aperture contour both relative to shell size, and to the extent of lateral shell compression. In a separate experiment, we found that snails raised in still water in a common garden showed higher resistance to water flow if originating from a wave environment than from a crab environment, and this was true both at juvenile (2 weeks) and adult (10 months) developmental stages. This result suggests genetic control of a distinct “wave adapted” phenotype, likely to be maintained under strong divergent selection between the two adjacent habitats.

## Introduction

A correlation between an environmental factor and a phenotypic trait is not sufficient to infer that there is local adaptation of the trait in response to that factor. Alternatively, it may be a by-product of adaptation at some other trait, or a consequence of a developmental constraint or plasticity, and it can prove difficult to disentangle the causative links between environment and phenotype [1]. On rocky shores under heavy wave exposure, one might expect that intertidal gastropods have shell shapes that are adapted to withstand hydrodynamic stress, which constitutes a strong threat to their survival. However, this is not always true. One example is the owl limpet (*Lottia gigantea*) that lives in a high energy wave environment, but has a long conical shell that is poorly designed to withstand rapid water flow [2]. In fact, waves are not an important selective factor on the shell shape of this species because it has evolved a sufficiently powerful adhesion system to resist strong water flow irrespective of shell shape. The case of the limpet illustrates the difficulty of linking a specific selection pressure to a specific phenotype. This is particularly difficult at environmental transitions where, in the same place, multiple environmental factors and many traits may change in concert. While trait-environment correlations may suggest a link, to determine whether a phenotype is truly responding to variation in a specific selection pressure needs experimental support.

**Fig 1:**
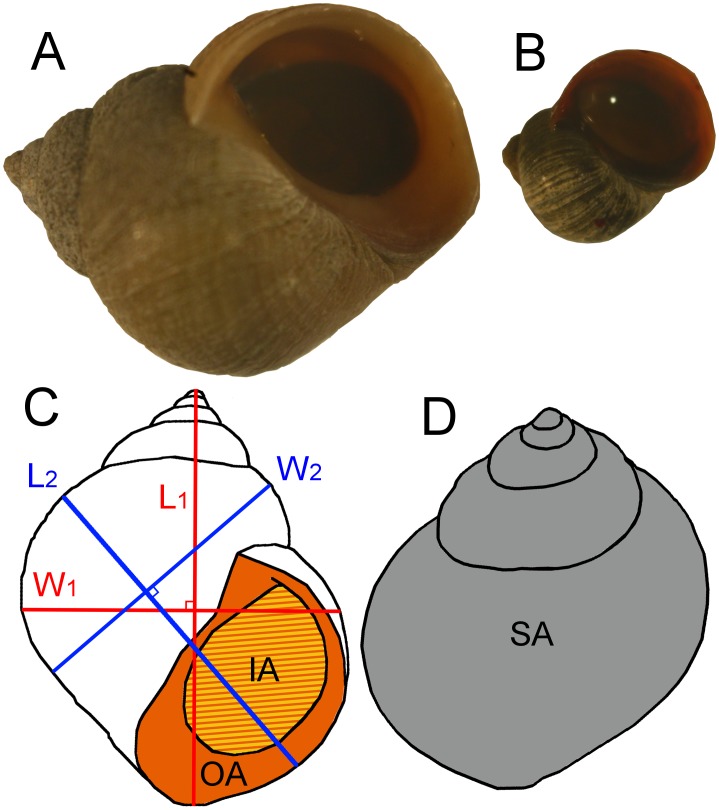
Crab and wave ecotype *L*. *saxatilis* showing measurements of the shell. A) Typical “crab” ecotype with large size, thick shell, wide aperture, and an elongated spire, sampled at left (boulder) end of the transect shown in Fig 2. B) Typical “wave” ecotype with small size, thin shell, wide aperture and compressed spire, sampled ~10 m before the right (cliff) end of the transect in Fig 2). C) Shell measurements (aperture view) used in this study: Outer aperture area (OA, colored orange), inner aperture area (IA, colored yellow), Length 1 (L_1_), Width 1 (W_1_), Length 2 (L_2_), Width 2 (W_2_). D) Projection of the shell area (SA, colored gray) from the spire view.

Populations of the marine snail *Littorina saxatilis* are excellent examples of biological systems where a multitude of phenotypic traits change simultaneously at environmental transitions. For example, a steep phenotypic change typically occurs between snails living in wave-exposed and in crab-rich microhabitats only meters apart [3–4]. The phenotypic divergence includes size at maturation, average adult size, shell shape, physiology and behaviour. Indeed, the two ecotypes are easily distinguishable, with the “crab ecotype” having a thick and elongated shell opened by a small aperture, and a large adult size (>7 mm, Fig 1A), while the "wave ecotype" forms a thin shell with a more or less compressed spire, a relatively large aperture, and a much smaller adult size (>2–3 mm, Fig 1B). In contact areas between different microhabitats, phenotypes diverge while connected by gene flow shaping steep phenotypic clines over the environmental transitions that are typically 10–30 m wide [3, 5, 6].

This divergence occurs repeatedly over very distant European sites, offering numerous cases of local phenotypic divergence over crab-wave environmental gradients [3–5, 7]. As *L*. *saxatilis* lacks a pelagic larva and dispersal is mostly limited to the life-time cruising range of a few meters [8, 9], these parallel phenotypic patterns are strong evidence that phenotypic divergence is maintained by local selection pressure.

The recent exploration of the molecular evolution of the system [10–14] has added to the understanding of the underlying genetic mechanisms, but thus far, uncertainty remains with respect to identifying the forces of selection that impose the repeated phenotypic pattern, and more specifically, the link between specific selection pressures and trait divergence [15]. Wave action has frequently been suggested as one major environmental factor that affects phenotypic differentiation [16–19]. Flow velocities and the associated hydrodynamic forces are greater when waves break on bare and steep rocky shores than on boulder beaches with shallow gradients [20]; on the other hand, boulder beaches are densely populated by crabs that are important predators on marine snails [21, 22].

Reciprocal transplants have demonstrated that differential selection is important in maintaining overall ecotype divergence [8, 17], and other studies have shown that crab predation selects for traits typical of the crab ecotype living in crab-rich areas, such as a large size, a thick shell and a small aperture [21]. Selection mechanisms that support formation of the contrasting ecotype, that is, the wave ecotype confined to wave-swept cliff environments, have been suggested [8,17, 23, 24] but incompletely investigated. For example, it has been suggested that the large aperture typical of the wave ecotype accommodates a comparatively larger foot and that the larger this area, the more a sea snail is resistant to dislodgement due to the waves sweeping the cliff surfaces [23, 25, 26, 27]. Variation in aperture area and size also affects vulnerability to desiccation and thermal stress, and variation in these traits may be linked to variable exposure in the two microhabitats [28]. Explanations for a smaller shell size include earlier maturation, and a size that allows for better exploitation of narrow crevices for protection on cliff surfaces [28, 29]. In addition, a specific shell trait may often be constrained by, and linked to other shell traits [1, 30–32] or emerge as a by-product of a faster growth rate [33]. Although the involvement of above mentioned factors is likely to play a role, direct measures of the resistance of Littorinids to dislodgement by water flows are lacking.

Thus experimental data are required to test whether the wave ecotype of *L*. *saxatilis* inhabiting the cliff environment has evolved adaptations to resist the stronger energies of water movements in a wave-exposed habitat and to better understand which traits are involved. In this study, we experimentally compared resistance to controlled hydrodynamic forces for individuals of *L*. *saxatilis* sampled at well-defined positions along an environmental gradient, which spanned a complete Swedish “crab” to “wave” habitat transition from a boulder bay to a cliff outcrop of smooth granite rock. To be able to make accurate measurements of individual resistance to hydrodynamic forces, we used a high-speed flume and measured resistance to a range of flow velocities for each snail. In addition, we measured each snail for traits including shell area, shell shape, foot area, and outer and inner aperture areas, to be able to relate variation in specific traits to flow resistance. While morphological and life history differences between ecotypes of *L*. *saxatilis* are for a large part heritable [29, 34], most quantitative shell traits have also shown plasticity to a certain extent in earlier studies [34–36]. To assess the degree of inheritance of traits involved in flow resistance we also raised juvenile and adult snails of both ecotypes in a common garden and measured their resistance to water flow in the high-speed flume.

## Material and methods

### Field collected snails from a hybrid zone

**Fig 2:**
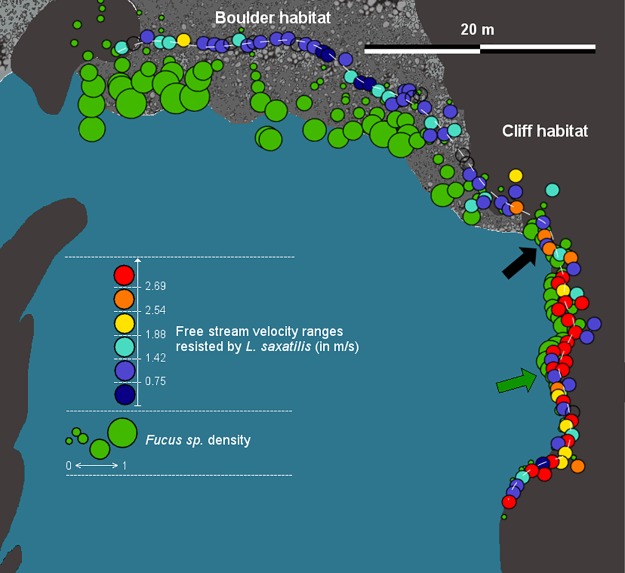
Resistance to water flow along the wave exposure transect. Schematic view showing the southern bay of the island of Ramsö (N 58°49'27", E 11°03'46"). The sampling position of each snail is represented by a circle filled with the colour indicating the snail's maximum resistance to water flow in the experimental pipe. Each colour is bounded by two free stream water velocities, except dark blue that shows maximum resistance < 0.75 m.s^-1^, and red that shows minimum resistance > 2.69 m.s^-1^. The sampling positions of the snails that did not attach to the flume pipe are marked by empty circles. Relative density of *Fucus* seaweeds is illustrated by green circles, wider circles representing larger amounts of *Fucus* (on a relative scale from 0 to 1) in an area of 4 m radius around the point. The Bézier path used for assigning a position to each snail in the clinal fit is represented by a dashed white path. A black arrow marks the transition from boulder to cliff habitat, a green arrow marks disappearance of *Fucus* sp.

In order to test resistance to water flow in snails living at different degrees of wave exposure, we sampled 100 *Littorina saxatilis* along a transect from a boulder bay (crab habitat) to a cliff outcrop (wave habitat) on the south shore of the island of Ramsö (N 58°49'27"; E 11°03'46") on the Swedish west-coast, in June 2015 (Fig 2). The spatial position (x,y,z) of each snail was taken with a precision of <1 cm using a total station theodolite (Trimble M3). Snails were brought to the laboratory and stored at +1°C in individually labeled tubes for a maximum of 3 days until their performance was assessed in the flume-flow experiment (described below). An index of fucoid (seaweed) coverage was used as an integrated estimate of wave exposure at different positions along the environmental transect [37–38]. In a separate survey of the same area, presence or absence of fucoid seaweeds in >150 small areas (10x10 cm) was scored and positioned along the same part of the shore using the total station. The coverage of fucoids along the shore was estimated by a weighted average of the presence (1) vs absence (0) scores with the weight being a linear function of distance from the focal point, declining from 1 at the focal point to 0 at 4 m from the focal point.

A Bézier path was calculated with the bezierCurve() function (rosettacode.org) in R to fit the x,y coordinates (z, height, was not included) of the sampled snails into a smooth transect (dashed line in Fig 2). The closest point on the curve for each snail sampling position was picked with the function locator() of R. This way we obtained a single variable (“position”) for each snail, describing its location on the transect. The position variable ranged from 0 at the boulder end to 76.25 m at the cliff end.

### Snails raised in a common garden

We sampled more than 50 adult female crab and wave ecotypes (but no intermediate phenotypes) at the extreme ends of two additional contact zones on two islands, Saltö (N 58°52'16'', E 11°7'11'') and Ramsökalv (N 58°50'4", E 11°2'27''). Adult females typically carry embryos in their brood pouches; these offspring were raised in standardized environmental conditions in the laboratory. In the population from Saltö we measured water resistance in the flume (see below) of the laboratory-raised offspring of each ecotype at two weeks age and at a size of 0.4–0.5 mm, while offspring of the Ramsökalv populations of crab and wave ecotypes were raised to adult age (10 month) and size (mean ± SD; crab: 5.6 ± 1.1 mm, wave: 4.9 ± 1.0 mm). Primarily we used these samples to assess plastic vs. inherited differences in resistance to water flow between the two ecotypes at juvenile and adult stages, but these samples also provided partial spatial replication of the analysis of the transect at Ramsö. University of Gothenburg, who performed the sampling and rearing of the snails, have a general permission to sample other than OSPAR listed or redlisted species in the Kosterhavet National Park. The permit is launched by the County Administrative Board of Västra Götaland, Sweden, with the number 521_42990–2014. *Littorina saxatilis* is not on the Swedish red list nor on the OSPAR list of endangered species.

### Measuring resistance to water flow using a high-speed flume

We constructed a pipe-flow flume where seawater is accelerated by falling from a 115-L head-tank filled to 75 L (1.0 m height difference). Upon release, the water flushes through a Plexiglas pipe (10 cm in diameter). The Plexiglas pipe can be opened and snails introduced to attach to the inner surface before each test. A controlling valve opens the flume to various degrees, thereby varying the water velocity in the pipe. Water speed in the pipe at five different standard positions of the valve was measured at different distances from the pipe wall with particle image velocimetry (PIV) (S1 File).

Adult snails were individually marked with nail polish, placed in the Plexiglas pipe, and acclimatized for 30 seconds to a gentle water flow. The small juvenile snails were treated in a similar way but could not be marked and we therefore ran each population separately. The acclimatization period allowed the snails to attach firmly and to orient facing the flow direction. No more than 5 snails were simultaneously tested to avoid snail interactions. Each group faced a series of 3 similar flow speeds lasting 4s each, with 30s resting time between each heat. The snails remaining in the pipe after the flushing sequence faced an increased speed for the following 3 flushes, and so on until all snails were either dislodged or had resisted the maximum water speed of the flume. For snails sampled along the transect, each individual went through the procedure twice, while for laboratory raised snails we ran one round of tests per snail. Adult snails that did not attach during the period of acclimation to flow (about 10% of the total, and mainly snails of the crab ecotype) were not included in down-stream analyses. However, due to the very small size of the juvenile snails we were not able to determine with certainty whether they were attached or not before the first test flush. Following Trussel [23], we used the maximum flow speed resisted by an individual during the two rounds in our analyses as the estimate of relevant “flow resistance” of that snail. The reported maximum flow speed that each snail resisted is expressed as the corresponding maximum free stream velocity recorded in the flume pipe. This is the maximum water speed at the center of the pipe at a given valve aperture angle. Adult snails should experience marginally lower flow speeds than this, but the small juvenile snails were affected by the boundary layer formed close to the substrate (S2 File) and experienced lower local speeds. However, we use the free stream velocity as a reference flow speed for all measurements, as it represents the speed of the moving waves.

### Shell and foot morphometry and statistical analyses

For the snails sampled along the transect only, aperture and spire views of the shell were photographed (Fig 1C and 1D). From these images, shell area (SA), outer (OA) and inner (IA) aperture areas (following [21]) and two different shell length measurements (L1 and L2, each with an orthogonal width measurement (W1 and W2) were all measured using the Image J software [39]. L1 was the distance from the tip of the spire to the most distant point of the shell, and its corresponding width (W1) was its maximum orthogonal distance. L2 was the maximum distance from the front (in a crawling motion) of the aperture to the back of the first coil, and W2 was the maximum distance orthogonal to L2 (Fig 1C). In addition, the underside of the foot for each snail during crawling on a transparent surface was photographed and the foot area (FA) was measured from this photograph (S1 Fig). The foot area of 13 snails could not be photographed (because the snails did not come out of the shell) and these individuals were discarded from down-stream morphometric analyses requiring foot area. The absolute values of foot and aperture areas were divided by the shell area to get size-independent estimates (relative foot area; RFA, relative outer aperture area; ROA, and relative inner aperture area; RIA). The two ratios L1/W1 and L2/W2 were used to indicate size-independent shell shapes of each snail (shape 1; S1 and shape 2; S2). Shell area (SA) was used as a proxy for size.

As we did not expect a linear relationship along the transect for the morphological traits nor for flow resistance of the snails, clines were fit for each morphological trait (RFA, ROA, RIA, S1, S2, SA). The cause of dislodgement is the water force exerted on a snail's surface and it scales to the square of the water velocity [40], the maximum free stream velocity variable was therefore squared before fitting a cline of flow resistance versus position on the transect. The simple cline function for a phenotypic trait used in [41] was fitted to traits and positions of individual snails by maximising the likelihood with the mle2() function of the package bbmle [42] in a custom R script. This cline function has seven parameters: centre, width, mean for the crab ecotype, mean for the wave ecotype and standard deviations for the crab, wave and “hybrid” areas. Initial values of these parameters were estimated by visualizing plots. A Chi-square test was used for comparison of the cline fit with a model of constant phenotype along the transect (taking 2 parameters: mean and sd), with χ^2^ = 2*(log likelihood of the cline model—log likelihood of the null model), and df = 5 [43]. The residuals from each cline fit, that is the difference between the measured value and the fitted value, were used as variables for further analysis. Traits that strongly contributed to the snails' capacity to resist water flow were expected to have residual correlation with the residuals of the water velocity. Consequently, we measured correlations for each trait’s residuals against the water velocity residuals (function rcorr() of the R package Hmisc option “pearson” and “spearman”). Shapiro's test showed deviation from normality of residuals of shell area, inner aperture area and shape 1. The spearman correlation coefficient was used for trait residuals that deviated from normality, otherwise, pearson correlation coefficient was used. To test the independence of the morphological traits, after allowing for clinal variation across the habitat transition, we also performed pairwise tests for correlations between residuals of the morphological traits.

For laboratory-raised juvenile and adult populations originating from Saltö and Ramsökalv, differences in maximum water flow resistance between offspring of crab and wave ecotypes were assessed with a Kruskal-Wallis rank-sum test (non-parametric, adapted to unequal sample size) with the function kruskal.test() provided in R. Figures were created in R with the package ggplot2() and Gimp 2 for further layering.

## Results

### Flow measurements

The free-stream flow velocity in the high-speed flume stabilized after roughly a second from opening of the valve and remained stable for at least 4s (S2 File). Both the acceleration of flow and the maximum flow velocity increased with increased level of valve opening. The maximum free-stream velocity reached was 0.75 m s^-1^ at level 1, 1.42 m s^-1^ at level 2, 1.88 m s^-1^ at level 3, 2.54 m s^-1^ at level 4 and 2.69 m s^-1^ at level 5. These velocities do not attain the maximal speeds that can exist in the field but cover most typical water speed conditions for breaking waves in the study area (S3 File). After the initial acceleration of flow there were cyclic events of deceleration followed by acceleration indicating the presence of large-scale eddies in the pipe (S2 File) similar to irregularities likely to be common in nature.

#### Snails' resistance to water flow along the transect

The transition from boulder to cliff habitat was accompanied by diminishing density of *Fucus* seaweed and, finally, its complete disappearance, suggesting increased levels of wave stress (Fig 2). Snails’ resistance to water flow in the high-speed flume changed over this transition with snails sampled towards the wave-exposed cliff habitat being more resistant than snails from the less-exposed side of the transect (Fig 2).

**Fig 3:**
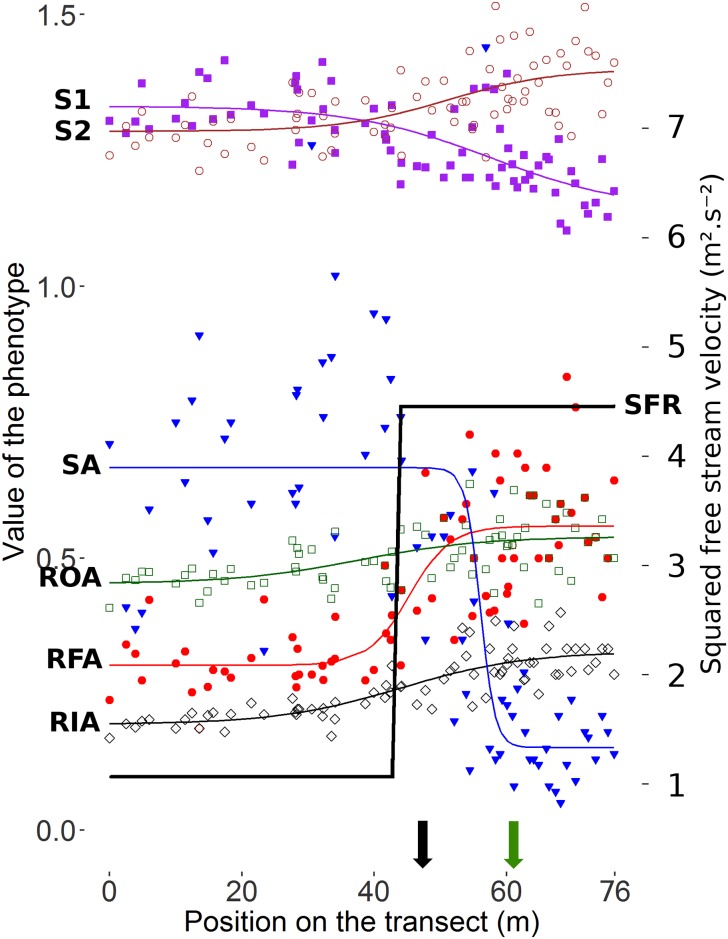
Variation in morphology along the transect. Shell shapes 1 and 2 (S1 and S2, as shown in Fig 1) estimate shell globosity and lateral compression, respectively, and foot area (FA), outer and inner-aperture areas (OA, IA, see Fig 1) were scaled on the shell area (SA) to represent relative areas (RFA, ROA and RIA, respectively). Lines are the best fitting sigmoid functions describing the clines in the traits, with y values on the left of the graph. S1 filled purple squares, S2 empty brown circles, RFA filled red circles, ROA empty green squares, RIA empty black diamond. The best fitting cline for the squared flow resistance (SFR) is plotted as a bold black curve, with y values on the right of the graph. A black arrow marks the position of the transition from boulder to cliff habitat and a green arrow indicates the disappearance of *Fucus* sp.

All morphological traits changed along the Bezier path (Fig 3 and S4 File). Shell area, relative foot area and relative outer and inner aperture areas increased towards the wave exposed habitat. Shell shape 1 (L_1_/W_1_) showed a decreasing trend towards the wave exposed habitats, indicating a more globose shape of the snails at the wave side of the transect, while shell shape 2 (L_2_/W_2_) showed the opposite trend suggesting a laterally compressed width, in a crawling direction, at the wave side of the transect (Fig 3).

**Fig 4:**
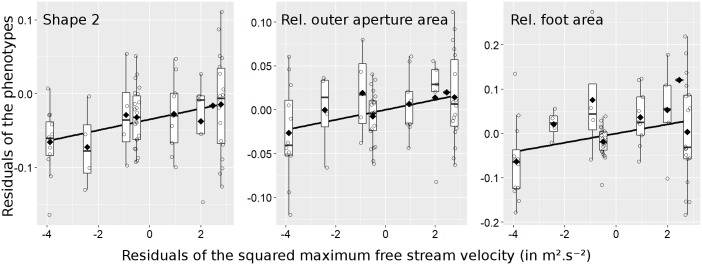
Boxplots of the phenotypes residuals vs the squared flow resistance residuals. Residuals of the three phenotypes with significant association with the snails' flow speed resistance are plotted against the residuals of squared maximum free stream velocity resisted. Boxes encompass 50% of the data points for each flow speed category. The median and mean values for each category are represented respectively as a thick horizontal bar and a filled diamond. The whiskers extend to the most extreme data point within 1.5 times the length of the box away from the box. Black regression lines illustrate the positive relationship between the trait residuals and flow resistance residuals. Small amounts of random variation were added in the x dimension to facilitate the visualization of all data points.

Among the six morphological traits measured, the residuals for three traits correlated positively with higher residual resistance to water flow: shape 2 (a more laterally compressed shell), relative outer aperture area, and relative foot area (Table 1, Fig 4). No correlation was found with the remaining traits. Effects of individual traits remained difficult to separate because all of the morphological variables showed pair-wise correlations with each other, even after removing the clinal effects (S1 Table).

**Table 1:** Correlations between residuals of morphological traits and residuals of squared maximum flow speed resistance.

Morphological trait	Correlation coefficient	p-value
Relative foot area (RFA)	r = 0.26	0.024 *
Relative outer aperture area (ROA)	r = 0.29	0.012 *
Relative inner aperture area (RIA)	ρ = 0.13	0.49
Shape 1 (S1)	ρ = -0.09	0.88
Shape 2 (S2)	r = 0.30	0.008 **
Shell area (SA)	ρ = -0.22	0.47

Residuals for each morphological variable and from the squared maximum flow velocity resisted by the snails were extracted from the fitted clines, n = 75. Pearson's correlation coefficient “r” is reported when the trait values did not deviate from normality, otherwise, Spearman correlation coefficient “ρ”is reported. The three p-values indicated as significant remained significant when corrected for multiple testing using the Benjamini and Hochberg test [44].

**Fig 5:**
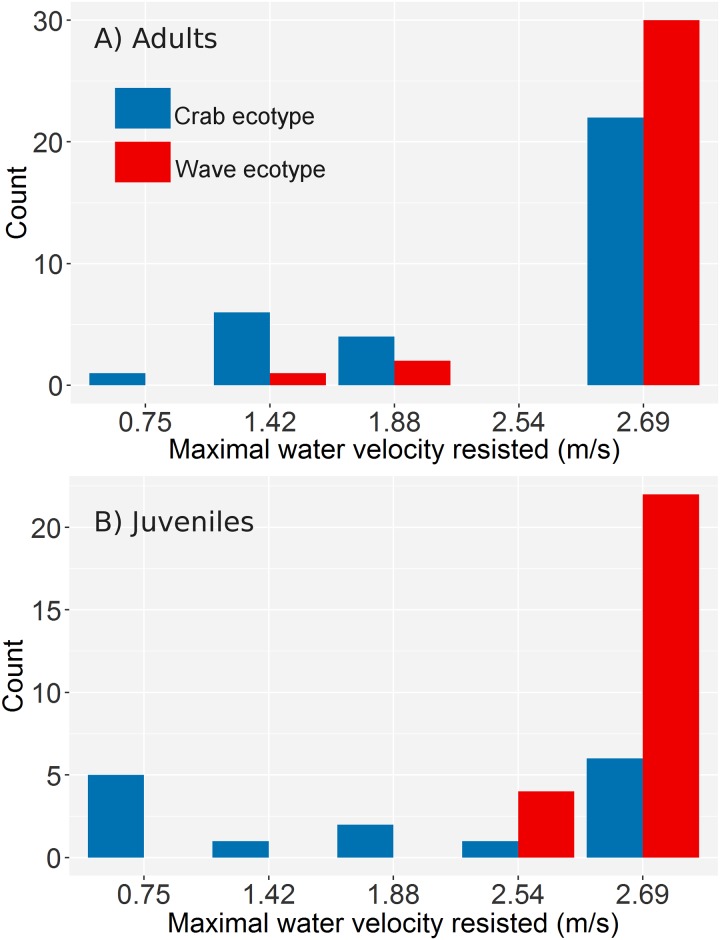
Resistance to water speed in snails reared in a common garden. Number of snails that resisted various maximum water speeds (free stream velocity). Note that the x-axis is not a linear scale. A) Ten months old adults born and raised in the laboratory from parents sampled at each end of a crab-wave transect (island of Ramsökalv). B) Two week old juveniles born and raised in the laboratory from parents sampled at each end of a crab-wave transect (island of Saltö).

Among adult snails raised in the laboratory common garden, 94% of the crab ecotype offspring and 100% of the wave ecotype offspring remained attached after exposure to the lowest flow velocity. Among the snails that remained attached, adult offspring of wave ecotype resisted water flow better than adult offspring of crab ecotype snails (Fig 5A and Kruskal-Wallis test: chi-squared = 4.43, df = 1, p = 0.035). Among the juveniles raised in the laboratory common garden only 33% of the crab ecotype offspring and 55% of the wave ecotype resisted a free stream velocity of 0.75 m/s. This may be due to the problem, described earlier, that we were unable to see if the tiny juveniles were properly attached or not before the water flow was turned on. Nevertheless, maximum resistance to water flow was higher in 2 week old juveniles of the wave ecotype than in similar age juveniles of the crab ecotype (Fig 5B and Kruskal-Wallis test: chi-squared = 11.46, df = 1, p = 0.0007).

## Discussion

Intertidal gastropods can reduce the risk of wave-dislodgement by at least three mechanisms. First, adaptive behaviours such as occupying crevices and a rapid switch to activity when submerged after a low tide to avoid being swept ashore by the first waves can reduce dislodgement risk [34]. Second, an appropriate body and shell morphology may reduce hydrodynamic drag and lift forces [25]. Third, an increased adhesive force exerted by the foot may reduce the risk of dislodgement [23, 25–27]. Hitherto, it has been shown that snails of the wave ecotype of *Littorina saxatilis* tend to seek shelter in crevices or similar structures in laboratory tanks [8], and are more active than snails of the crab ecotype [34] matching the expectation of a behaviour appropriate to wave-exposed conditions. In the current study, the second and third mechanisms that may further contribute to a lower risk of dislodgement in wave-exposed habitats were assessed experimentally.

Over the boulder to cliff transect, the progressive disappearance of *Fucus* seaweeds indicated increased wave action [37–38] and at the same time our experiments showed a larger resistance to hydrodynamic forces among the snails from the cliff outcrop than among those from the boulder habitat. Furthermore, we showed that such resistance was higher in snails with a larger foot area and a wider outer aperture area, largely supporting suggestions made earlier without experimental support [19, 23, 25, 27]. Additionally, we found that snails facing a flow with a shell more laterally compressed had higher resistance than snails that were wider when facing the flow—but note that the advantage provided by specific shell proportions is difficult assess due to correlations with other shell characteristics (S1 Table). For a given height, shells with a high S2 value have a smaller cross-sectional area towards the water flow, when oriented with the head towards the flow, and this orientation was preferred by the snails as soon as they experienced the first gentle flow of water. One additional observation made during the experiments with the flume was that the shell of the snails from the crab ecotype vibrated considerably more while exposed to the rapid flow than the shell of the wave ecotype snails. This suggests that shell shape of the wave ecotype delays flow separation and reduces turbulent eddy generation.

We observed that size and relative foot area had the steepest clines along the transect, while clines for the two shape variables and the inner and outer aperture areas were much more shallow (Fig 3). Interestingly, the size and foot area clines did not overlap in position; the center of the size cline was shifted towards the cliff end of the transect and coincided approximately with the disappearance of the *Fucus*, while the center of the foot area cline coincided with the transition from boulder to cliff substrate (S4 File). Multiple effects may cause clines not to overlap. For example, this could reflect that different selective forces may transition at different locations, or that the strength of opposing selective forces is asymmetric. One hypothesis is that if crab selection is primarily on shell size and thickness, the shell size decreases abruptly when *Fucus* disappears, where the shore crabs mostly hide (pers. obs. KJ) [21]. Foot area would increase at the boulder to cliff transition, due to the more dangerous hydrodynamic forces on cliffs. Indeed, even though snails may be dislodged by crabs in the boulder habitat they are likely caught among the boulders and remain in their habitat. On the cliffs however, dislodgement will most likely result in the displacement of the snail into the sublittoral zone where live *L*. *saxatilis* are not recorded. A second hypothesis is that selection by crab predation favouring big shells is stronger than the threat of wave dislodgement at the transition, and pushes the center of the size cline toward the cliff habitat, while affecting foot area less. Repeated analyses of clines in other contact zones are needed to corroborate or refute these suggestions, but preliminary results suggest that the size cline is most often displaced towards the cliff habitat (pers. obs. RKB and AMW).

Many studies indicate that organisms in wave-swept environments are limited in size [40]. This observation may suggest that shell size affects the ability of snails to cope with water flow, especially if smaller individuals benefit sufficiently from reduced velocities deep in the boundary layer. Nonetheless, even if smaller organisms expose a reduced area to the flow and thereby reduce the drag force, they also have a comparably smaller foot to hold on to the substratum. Consequently, for isometric shapes there may be no size difference in the resistance to flow other than the benefit of the boundary layer, with negligible effect on adult snails (but see below for juveniles). This prediction was supported in our experiments and, similar to [28], we found that, although size (as approximated by a spire view of the total shell area, SA) was strongly reduced among the adult snails from crab to wave ecotype in the studied transect of Ramsö, it had no significant effect on snail resistance to water flow. However, a smaller size will increase access to small crevices in the wave habitat and, in this way, facilitate the sheltering of snails from wave forces and other types of stressors, such as heating and desiccation [28, 29].

Surprisingly enough, the small juvenile snails raised in the laboratory performed very well under rapid flows, with velocities resisted matching those tolerated by adults of the same ecotype. Due to their tiny sizes (0.4–0.5 mm) the juveniles were in the boundary layer and therefore experienced 30–45% lower local flow speeds than the adult snails (speed estimates described in S2 File). Since the flow-induced drag and lift forces on a snail scale with the square of the local flow velocity around the snail [40], the very small juvenile snails were thus exposed to much lower forces than the adult snails, which contributed to their high capability to resist similar free stream velocities.

Offspring of wave and crab ecotype snails from two other islands had also different water flow resistance. This ecotype specific difference was found between juveniles and between adults snails raised in still water in the laboratory. Firstly, this shows that the pattern found in the first island sampled was not unique but was general to this type of environmental transitions and ecotype contact zones. Secondly, it indicates that the differences in flow resistance observed are largely heritable (even though our study design does not exclude maternal effects). Finally, it shows that this difference is observable very early in the life of the snails and that it persists in life even in absence of environmental stimuli.

Already in the range of 0 to 2.69 m.s^-1^ water velocity, differences between snails inhabiting boulders and snails inhabiting cliffs were clearly measurable. Nonetheless, the upper limit reached in the flume are flow speeds that are not excessively rare in the field, especially in the cliff habitat (S3 File). One hypothesis is that, since these violent events can induce high mortality in intertidal communities [45], they are strong selective events for the phenotypes that favor intertidal snails' resistance to dislodgement [18, 46]. Years with storms induced a significant increase in aperture areas in a population of *Littorina striata* living on an exposed site compared with years without storms [46]. In *Littorina obtusata*, an exceptionally strong storm was found to reduce mean shell height and also, counter-intuitively, to reduce mean aperture area [18]. If intertidal snails occasionally face these powerful events, it is to be noted that in our study, no snail collected in the boulders could withstand a speed of 2.69 m.s^-1^, and that a fraction of the snails collected on the cliff dislodged at lower speeds. In the present experiment, the snails were placed in a flume without structures that slowed down the water flow, and therefore exposed to water velocities that are close to the free stream velocity. In the field, snails will probably avoid the maximum speeds of breaking waves by finding refuge in cliff crevices or among boulders and mitigate physical stress of storms. The comparison of the measurements obtained experimentally with the conditions that can exist in the field suggests that the snails' use of the microenvironment to reduce local flow speed is crucial to maintaining their grip in stormy conditions.

Our results support the expectation that a common intertidal gastropod, *Littorina saxatilis*, is locally adapted to withstand strong water forces in populations inhabiting cliff surfaces where snails are from time to time heavily exposed to waves. Traits most involved in this adaptation are foot area, the outer shell aperture area, and the lateral compression of the shell shape, while size seems not to play a role in resisting rapid water currents. The adaptive phenotypic components were shown to be heritable and effective already in 2-week-old hatchlings.

## Supporting information

S1 Dataset (XLSX)

S1 File Description of the high-speed flume. (DOCX)

S2 File Description of flow measurements. (DOCX)

S3 File Wave model based on wind speed, fetch and depth. (DOCX)

S4 File Summary of the maximum likelihood cline fits. (DOCX)

S1 Table Correlations of morphological traits. (DOCX)

S1 Fig Picture of foot area. (TIFF)

S2 Fig Correlations between morphological measurements. (TIFF)
