# Supplementary material for: Adaptation to dislodgement risk on wave-swept rocky shores in the snail Littorina saxatilis
Source: PLoS One. 2017 Oct 23;12(10):e0186901. doi: 10.1371/journal.pone.0186901 (PMC5653359; doi:10.1371/journal.pone.0186901)

**S1 File: Description of the high-speed flume.**

Drawing of the high-speed pipe-flow flume used in the flow resistance experiments. All dimensions are given in millimetres. The seawater in the pipe-flow flume is rapidly accelerated from a head-tank through a 100 mm (inner diameter) pipe. The water velocity in the pipe can be controlled with a valve at the downstream end of the pipe. The 115-L overhead tank is filled by pumping water from the reservoir (and outflow) tank. An overflow pipe mounted in the overhead tank drains water back into the reservoir tank and ensures a constant pressure head (height difference) of 1.0 m before the controlling valve is opened. Illustration: Thore Hilmersson


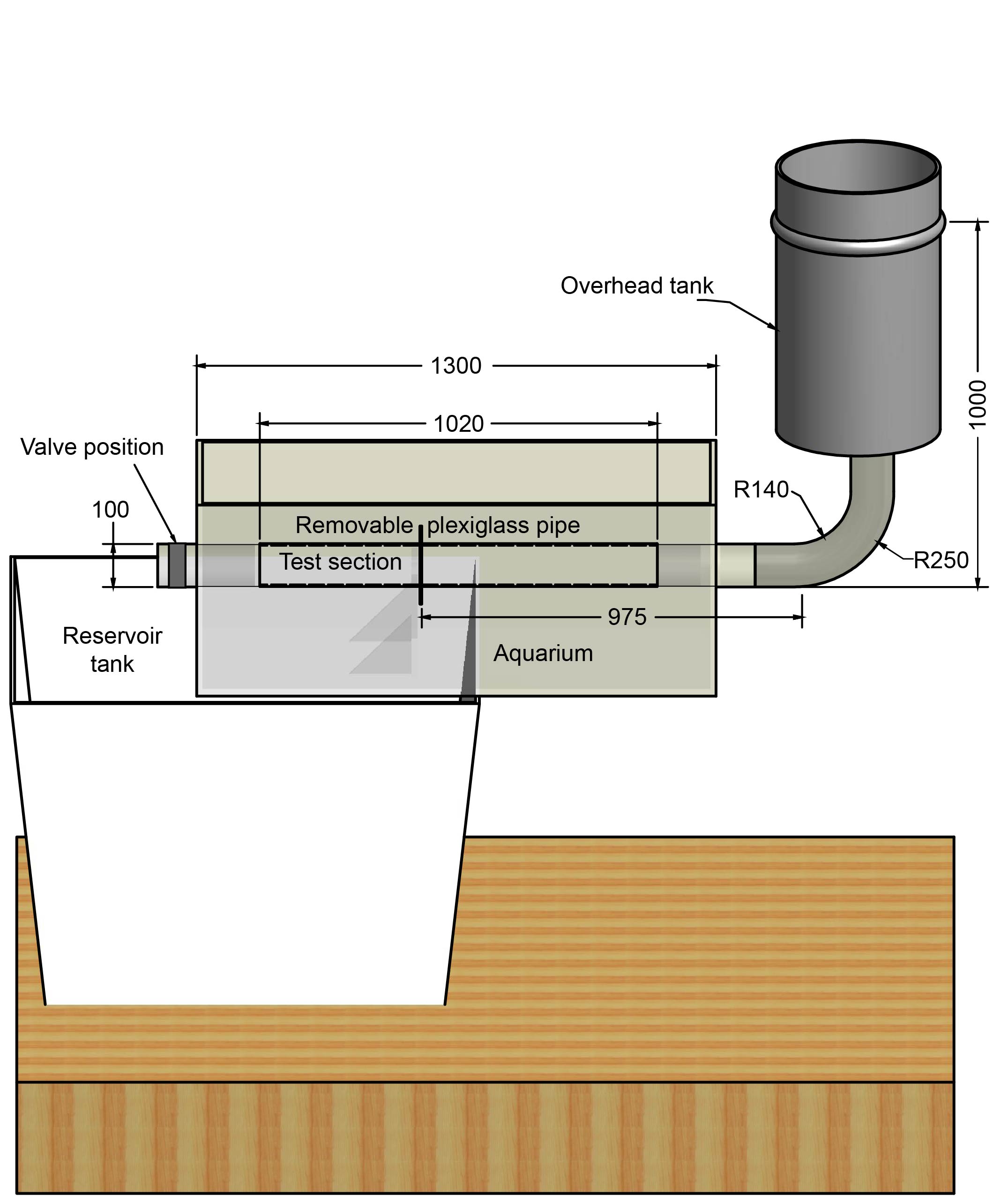

Supplement: S1 File — (DOCX) [file pone.0186901.s002.docx]
