# Supplementary material for: Adaptation to dislodgement risk on wave-swept rocky shores in the snail Littorina saxatilis
Source: PLoS One. 2017 Oct 23;12(10):e0186901. doi: 10.1371/journal.pone.0186901 (PMC5653359; doi:10.1371/journal.pone.0186901)
Supplement: S2 File — (DOCX) [file pone.0186901.s003.docx]

S2 File: Description of flow measurements

Particle image velocimetry (PIV) was used for measurements of flow speed in the high-speed flume. The seawater in the system (20°C, 33 psu) was seeded with 10-µm glass spheres as tracing particles. Measurements took place in the test section at the downstream end of the Plexiglas pipe (Fig. S1). The pipe flow was illuminated from above with a double-pulsed Nd:YAG laser (Litron, 30 mJ at 532 nm). The beam was expanded by a combination of lenses into a 1-mm thick vertical light sheet aligned parallell to the flow direction at the centre of the pipe. The PIV-camera (LaVision Imager Pro X, 1600×1200 pixels) with a Nikon 50-mm Nikkor lens and a 532 nm bandpass filter was run in the double-frame mode. Recordings were done at 10 Hz covering a distance of 9 cm in the streamwise direction and from the pipe floor to the pipe centre in vertical direction. Measurements were made at five different standard positions of the controlling valve. Recorded images were analysed with the DaVis 8.2.0 software (LaVision) using cross correlation and a final interrogation window size of 16×16 pixels. A 50% overlap between interrogation windows resulted in a resolution of 22 flow vectors per cm. Due to laser reflection at the pipe floor, reliable flow vectors were obtained no closer than 0.96 mm from the floor.

For each instantaneous measurement, the streamwise flow vector components were extracted for heights from 0.96 to 15 mm above the pipe floor and for 50 mm above the floor representing the free-stream flow in the centre of the pipe. For each height, the average flow velocity was calculated from all vectors along a 9-cm downstream stretch of the pipe. Figure A shows flow velocities measured at 50 mm above the pipe floor.

For comparisons of flow-induced forces between juvenile-sized and adult-sized snails, local flow speeds in the pipe boundary layer were used. These local flow speeds were extracted at the time (for each flow level) when flow speed in the pipe was at the maximum. Local flow speeds closer to the pipe floor than could be measured by PIV (*z* < 0.96 mm) were derived from applying the “law of the wall”. The mean local velocity in a turbulent boundary layer is expected to grow with the logarithm of the distance from the substratum and over a relatively smooth surface the following equation is used to describe the velocity gradient [40]:


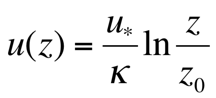
 eq. 1

where *u(z)* is the mean velocity parallell to the substratum at a distance *z* above the surface, *u*_*_ is the friction velocity, *κ* is von Karman’s constant and *z_0_* is the roughness parameter. The measured local velocities were plotted against the logarithm of the distance from the pipe floor and a linear expression was fitted (Fig. B). The linear regression was extrapolated to obtain local velocities closer to the floor than could be measured. The boundary layer was assumed to be logarithmic all the way down to the very thin laminar sublayer (< 160 µm for all flow speeds).


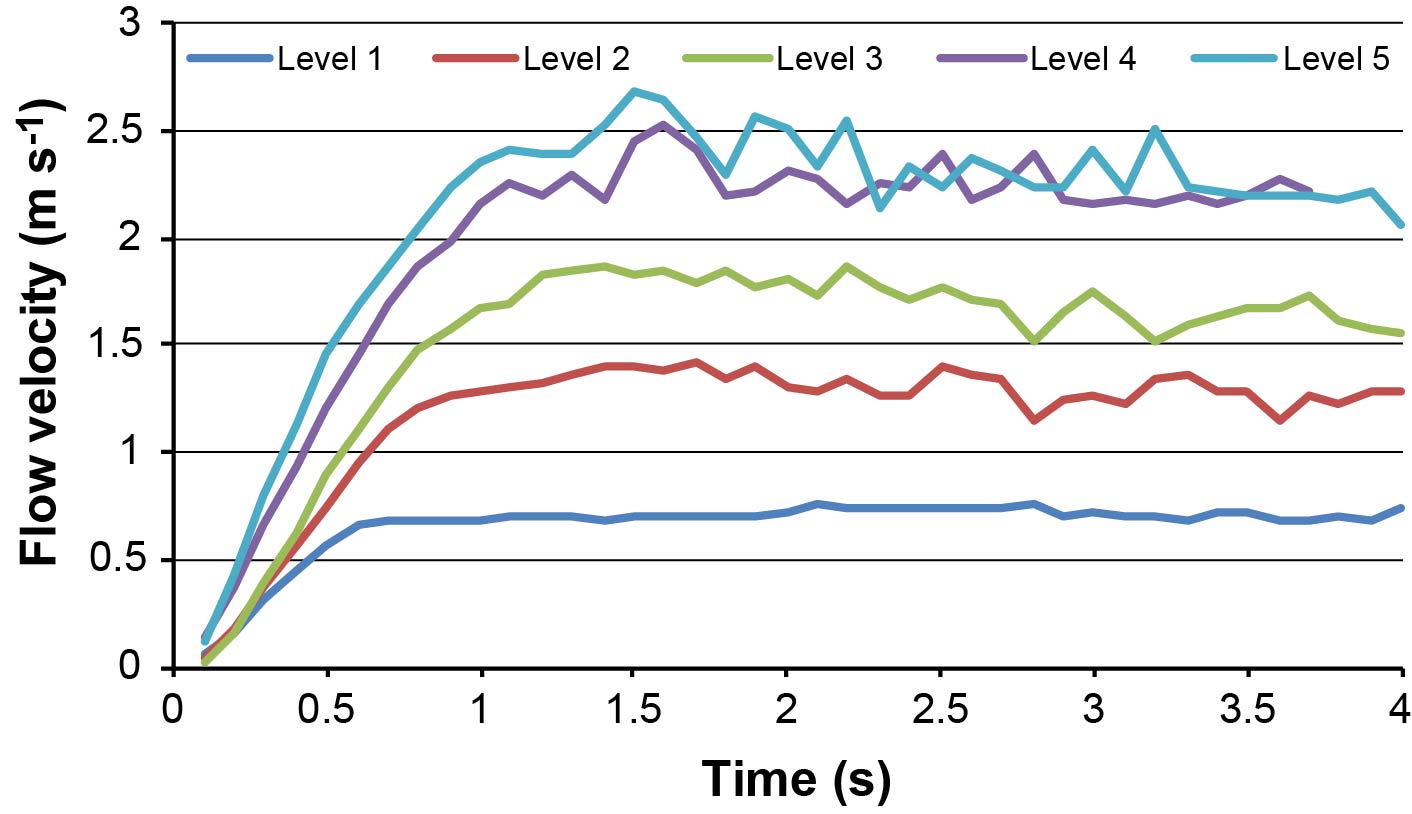


**Figure A.** Flow velocities measured along the centre axis of the high-speed flume pipe after opening of the controlling valve. The valve was opened in 5 different standard steps (level 1-5) resulting in increasing flow speed.


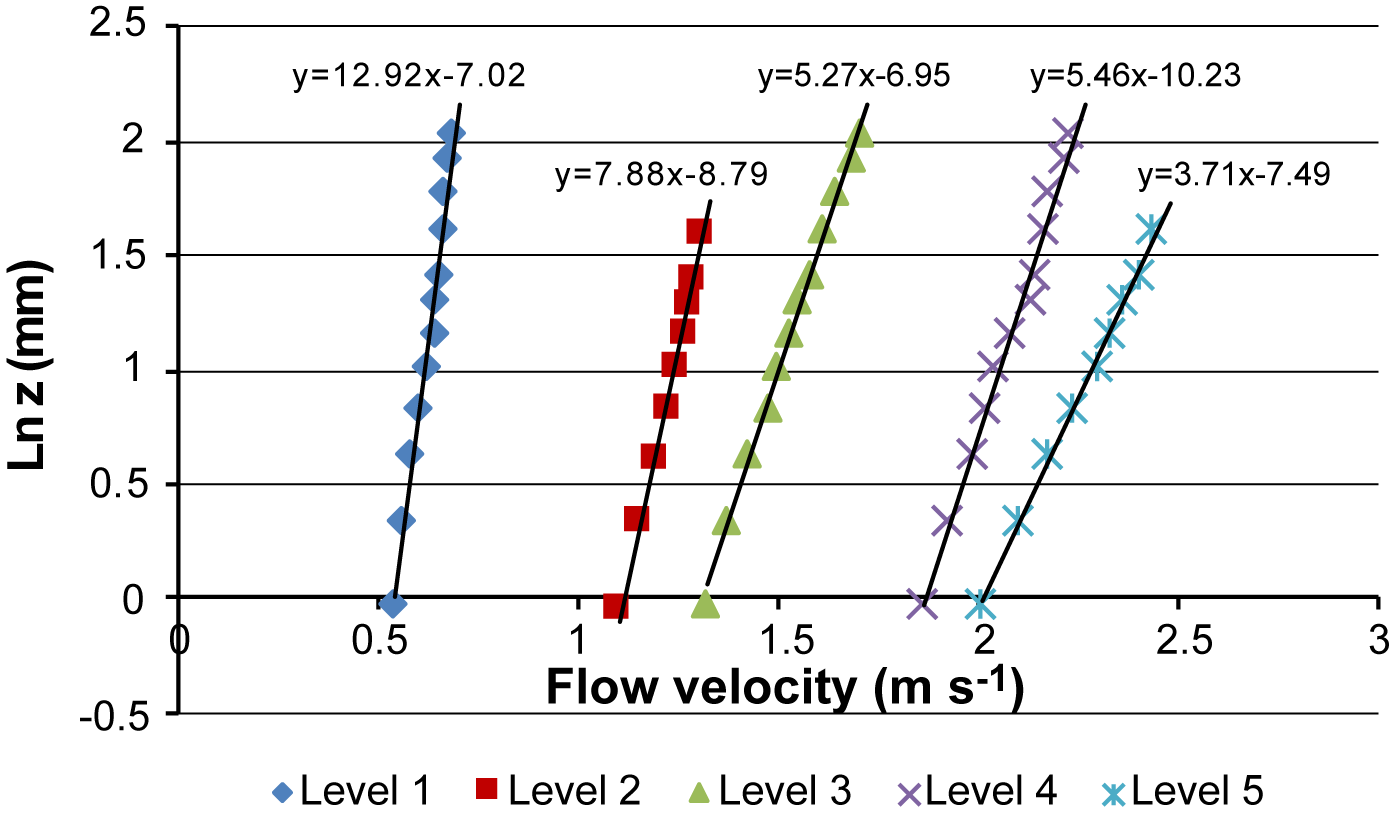


**Figure B.** Velocity profiles measured within the boundary layer of the high-speed flume pipe floor. For each step of the valve opening (level 1-5), velocity profiles were extracted at the time when flow speed in the pipe was at the maximum. The equations describe the linear fits (R^2^ > 0.98 in all cases) between the velocity close to the floor and the logarithm of the distance from the floor (*z*).
