# Supplementary material for: Adaptation to dislodgement risk on wave-swept rocky shores in the snail Littorina saxatilis
Source: PLoS One. 2017 Oct 23;12(10):e0186901. doi: 10.1371/journal.pone.0186901 (PMC5653359; doi:10.1371/journal.pone.0186901)
Supplement: S3 File — (DOCX) [file pone.0186901.s004.docx]

**S3 File : Wave model based on wind speed, fetch and depth**

The maximum flow speed in breaking waves at the field locations where individuals of *Littorina saxatilis* were sampled was estimated with a fetch-based wave model. This simple model is based on the wind fetch, water depth, and hourly wind velocity measurements. The model is described in detail in the US Coastal Engineering Research Center (1984) and consists of two main equations for significant wave height and period, respectively (eqs. 2 and 3):

$\frac{gH}{U_{A}^{2}}=0.283tanh\left[ 0.53\left( \frac{gd}{U_{A}^{2}} \right)^{3/4} \right]tanh\left\{ \frac{0.00565\left( \frac{gF}{U_{A}^{2}} \right)^{1/2}}{tanh\left[ 0.53\left( \frac{gd}{U_{A}^{2}} \right)^{3/4} \right]} \right\}$ eq. 2

$\frac{gT}{U_{A}}=7.54tanh\left[ 0.833\left( \frac{gd}{U_{A}^{2}} \right)^{3/8} \right]tanh\left\{ \frac{0.0379\left( \frac{gF}{U_{A}^{2}} \right)^{1/3}}{tanh\left[ 0.833\left( \frac{gd}{U_{A}^{2}} \right)^{3/8} \right]} \right\}$ eq. 3

where *H* is the significant wave height, *T* is the wave period, *F* is wind fetch, *d* is water depth, *g* is the gravity constant, and *U_A_* is wind stress. Wind speed was converted to wind stress (*U_A_*) using the relationship (US Coastal Engineering Research Center 1984):

*U_A_* = 0.71**wind speed*^1.23^ eq. 4

The model predicts the significant wave height and this is multiplied by 1.5 to get an estimate of maximum wave height in a given wave field (Denny 1995). It is assumed that these waves break onto the rocky shore and give rise to a maximum flow speed, *U_max_*, as:

$U_{max}=3.75H_{b}^{0.57}$ eq. 5

Where *H_b_* is the height of the breaking wave (Denny 1995).

The wind data forcing the model were hourly means of wind speed and direction in sectors of 10° and cover three full years (2013-2015) collected by the Swedish Meteorological and Hydrological Institute (SMHI) from the Nordkoster weather station (58°53’N, 11°E).

We considered the “crab” and “wave” parts of the shore on Saltö (58°52’N, 11°7’E). The fetch in 10° sectors was measured from Google Earth. The wind direction from the wind time series (2013-2015) was matched to a fetch sector and the wind speed was used to calculate the significant wave height for that hour. The depth around the “crab” shore was set to 5 m and to 20 m for the “wave” shore. The maximum flow speed in breaking waves during one hour was calculated for each hourly wind speed datum for all three years. The percentage of time experiencing flow speeds between 0-8 m/s was then plotted for both types of shores (Fig. A). All calculations were made with Matlab R2016a (MathWorks, Inc). For the sheltered shore the maximum flow speed was below 1 m/s for 50% of the time. For the moderately exposed shore the 50% break point occurred at 1.9 m/s.

**Figure A: Frequency of different wave-speed in “crab” and “wave” environments in Saltö**

**Figure A:** Percentage of time during 2013-2015 that the sheltered “crab” and the exposed “wave” shores were exposed to breaking waves with maximum flow speeds between 0-8 m/s. Note that the proportion of time when there is no wave action was 68% for the “crab” shore and 24% for the “wave” shore.

**References**

Coastal Engineering Research Center. 1984. Shore Protection Manual. U.S. Army Corps of Engineers, Waterways Experiment Station, Vicksburg Mississippi.

Denny M. Predicting physical disturbance: mechanistic approaches to the study of survivorship on wave-swept shores. 1995. Ecological Monographs 65: 371-418

Vogel S. Life in Moving Fluids. 1994. The Physical Biology of Flow. Princeton University Press, Princeton.
