# Supplementary material for: Adaptation to dislodgement risk on wave-swept rocky shores in the snail Littorina saxatilis
Source: PLoS One. 2017 Oct 23;12(10):e0186901. doi: 10.1371/journal.pone.0186901 (PMC5653359; doi:10.1371/journal.pone.0186901)
Supplement: S4 File — (DOCX) [file pone.0186901.s005.docx]

1. **S4 File: Summary of maximum likelihood cline fits**
2. **Table A: Parameters of the clines with maximum likelihood fits**

| 1. Fitted data | 1. Centre | 1. Width | 1. Mean | | 1. SD | | | 1. Log-likelihood 2. difference (Cline – Null) | |
| --- | --- | --- | --- | --- | --- | --- | --- | --- | --- |
|  |  |  | 1. Crab | 1. Wave | 1. Crab | 1. Hybrid | 1. Wave | |  |
| 1. Rel. foot | 1. 45.29 | 1. 12.36 | 1. 0.30 | 1. 0.56 | 1. 0.05 | 1. 0.03 | 1. 0.11 | | 1. +45.3 |
| 1. Rel. outer ap. | 1. 40.36 | 1. 47.42 | 1. 0.45 | 1. 0.55 | 1. 0.02 | 1. 0.02 | 1. 0.06 | | 1. +21.1 |
| 1. Rel. inner ap. | 1. 42.90 | 1. 22.83 | 1. 0.20 | 1. 0.32 | 1. 0.02 | 1. 0.01 | 1. 0.04 | | 1. +54.7 |
| 1. Shape 1 | 1. 57.76 | 1. 42.43 | 1. 1.33 | 1. 1.14 | 1. 0.04 | 1. 0.05 | 1. < 0.01 | | 1. +26.4 |
| 1. Shape 2 | 1. 57.33 | 1. 64.42 | 1. 1.27 | 1. 1.45 | 1. 0.03 | 1. 0.04 | 1. 0.07 | | 1. +20.7 |
| 1. Shell area | 1. 56.01 | 1. 4.44 | 1. 0.67 | 1. 0.15 | 1. 0.20 | 1. 0.47 | 1. 0.05 | | 1. +52.2 |
| 1. (Flow resistance) ² | 1. 43.31 | 1. 0.17 | 1. 1.06 | 1. 4.45 | 1. 0.78 | 1. 1.52 | 1. 2.92 | | 1. +39.4 |

1. Clines were fitted to the data using maximum likelihood estimation. The table summarizes the
2. position of the centre, and the width of the cline (in m). Mean phenotypic value at the crab and
3. wave ends of the cline and standard deviation (SD) at the crab, wave and hybrid zones. The
4. difference in log-likelihood of the fitted cline (7 parameters: centre, width, mean crab, mean wave,
5. sd crab, sd hybrid, sd wave) with the log-likelihood of a model of constant phenotype (“Null”
6. model taking 2 parameters: mean and sd of the phenotype) are reported. All clines fitted the data
7. better than the null model did, with χ² estimated p < 1.10^-4^ .
   - 1. Table B: Confidence intervals of the cline fits
     2. Limit values at 95% confidence interval for the parametres used in the maximum likelihood cline
     3. fits. Values were obtained with the confint() function of R.

|  | Relative foot area | | Relative outer aperture area | | Relative Inner aperture area | | Shape 1 | |
| --- | --- | --- | --- | --- | --- | --- | --- | --- |
| Conf. limit | 2.5 % | 97.5 % | 2.5 % | 97.5 % | 2.5 % | 97.5 % | 2.5 % | 97.5 % |
| Centre | 42.30 | 51.41 | 9.36 | 75.22 | 38.32 | 58.15 | 48.33 | 137.46 |
| Width | 3.85 | 32.48 | 19.07 | NA | 17.89 | 93.46 | 22.20 | 144.66 |
| Mean crab | 0.28 | 0.32 | 0.33 | 0.47 | 0.16 | 0.21 | 1.31 | 1.3 |
| Mean wave | 0.52 | 0.63 | 0.52 | 0.99 | 0.31 | 0.42 | 0.73 | 1.19 |
| S.d. crab | 0.04 | 0.07 | NA | 0.03 | NA | 0.02 | NA | 0.06 |
| S.d. hybrid | NA | 0.14 | NA | 0.05 | NA | 0.03 | NA | 0.26 |
| S.d. wave | 0.09 | 0.15 | NA | 0.41 | NA | 0.07 | NA | 0.48 |

|  | Shape 2 | | Shell area | | Squared flow resistance | | |
| --- | --- | --- | --- | --- | --- | --- | --- |
| Conf. limit | 2.5 % | 97.5 % | 2.5 % | 97.5 % | | 2.5 % | 97.5 % |
| Centre | 16.08 | 120.60 | 54.19 | 57.60 | | NA | NA |
| Width | 9.60 | NA | 2.23 | 8.89 | | NA | 6.93 |
| Mean crab | 1.13 | 1.30 | 0.60 | 0.73 | | 0.78 | 1.35 |
| Mean wave | 1.37 | 1.73 | 0.13 | 0.18 | | 3.56 | 5.35 |
| S.d. crab | NA | 0.05 | 0.16 | 0.26 | | NA | NA |
| S.d. hybrid | NA | 0.33 | NA | 1.11 | | NA | NA |
| S.d. wave | NA | 2.78 | 0.04 | 0.08 | | 2.40 | 3.67 |
