# Supplementary material for: Adaptation to dislodgement risk on wave-swept rocky shores in the snail Littorina saxatilis
Source: PLoS One. 2017 Oct 23;12(10):e0186901. doi: 10.1371/journal.pone.0186901 (PMC5653359; doi:10.1371/journal.pone.0186901)
Supplement: S1 Table — (DOCX) [file pone.0186901.s006.docx]

- - 1. S1 Table: Correlations of morphological traits
    2. Pairwise correlations between the residuals of the morphological traits from the fitted clines. Expecting a linear relationship we used Pearson's test, n=75, using the function rcorr() of the R package Hmisc option “pearson”). Above the diagonal are the correlations (Pearson's r) and below are the corresponding p values. Abbreviations of morphological traits as in Table 1.

|  | Res. RFA | Res. ROA | Res. RIA | Res. S1 | Res. S2 | Res. SA |
| --- | --- | --- | --- | --- | --- | --- |
| Res. RFA | --- | 0.43 | 0.25 | -0.45 | 0.39 | -0.31 |
| Res. ROA | 0.0001 | --- | 0.48 | -0.42 | 0.69 | -0.27 |
| Res. RIA | 0.0292 | 0.0000 | --- | -0.30 | 0.42 | -0.35 |
| Res. S1 | 0.0000 | 0.0002 | 0.0092 | --- | -0.51 | 0.32 |
| Res. S2 | 0.0005 | 0.0000 | 0.0002 | 0.0000 | --- | -0.27 |
| Res. SA | 0.0073 | 0.0179 | 0.0020 | 0.0045 | 0.0205 | --- |
