# Supplementary figures and images for: Adaptation to dislodgement risk on wave-swept rocky shores in the snail Littorina saxatilis
Source: PLoS One. 2017 Oct 23;12(10):e0186901. doi: 10.1371/journal.pone.0186901 (PMC5653359; doi:10.1371/journal.pone.0186901)

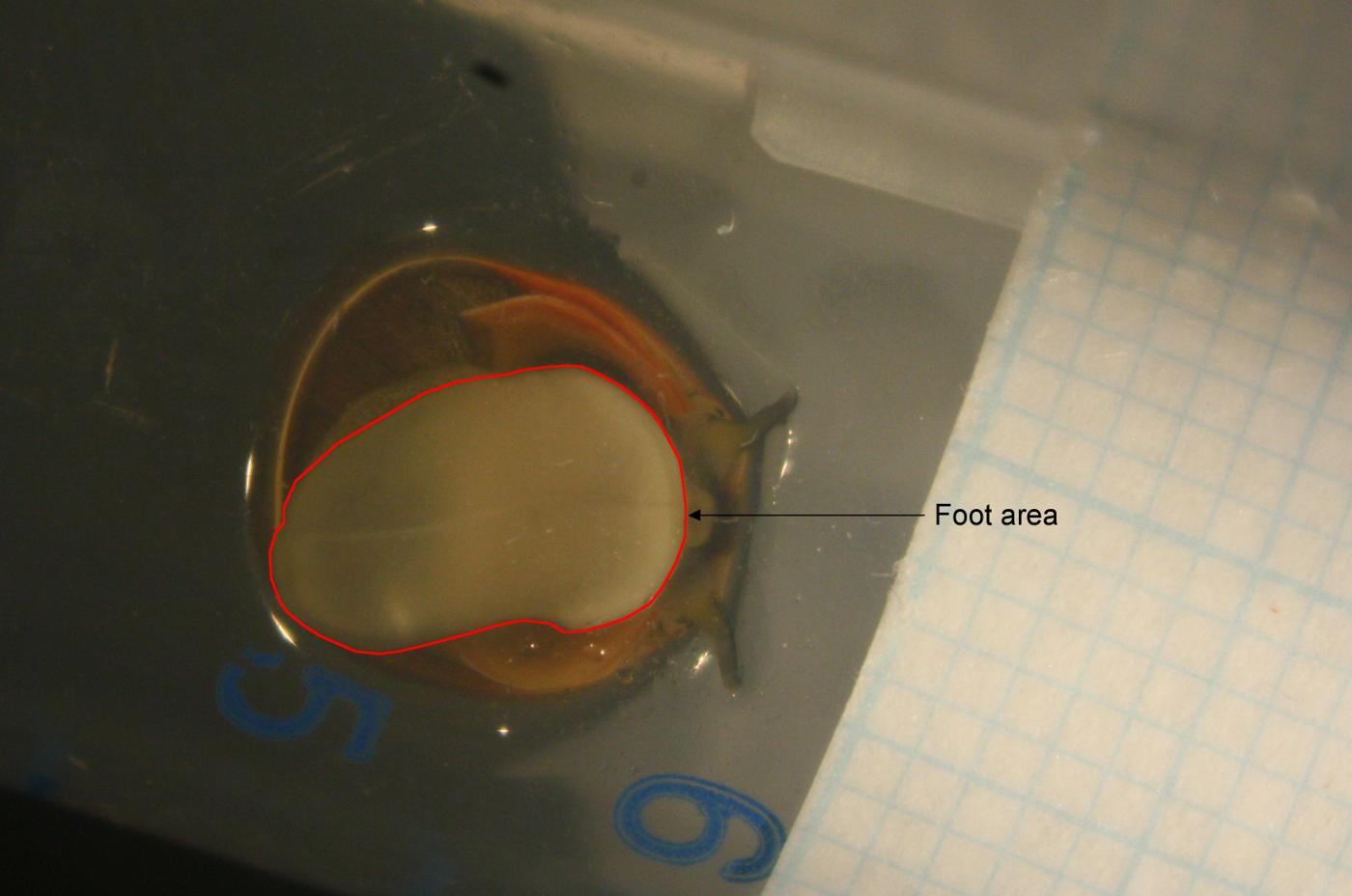

Supplement: S1 Fig — (TIFF) [file pone.0186901.s007.tiff]

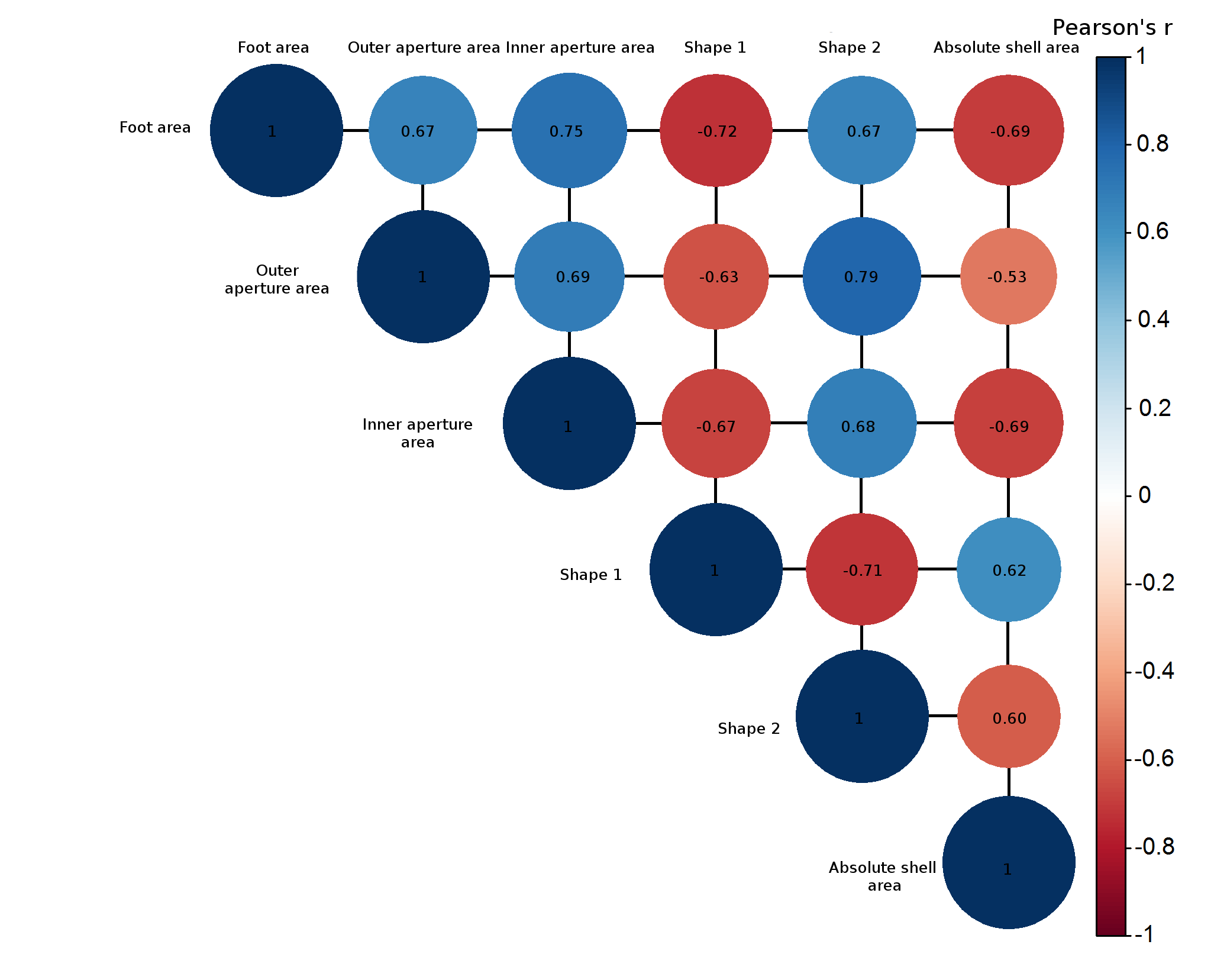

Supplement: S2 Fig — (TIFF) [file pone.0186901.s008.tiff]
